# Information, partisanship, and preferences in a pandemic

**DOI:** 10.3389/fpubh.2023.1019206

**Published:** 2023-03-08

**Authors:** Jonathan T. Rothwell, Christos Andreas Makridis, Christina Michelle Ramirez, Sonal Desai

**Affiliations:** ^1^Gallup, Washington, DC, United States; ^2^Institute of Public Policy, George Washington University, Washington, DC, United States; ^3^Metropolitan Policy Program, Brookings Institution, Washington, DC, United States; ^4^Chazen Institute in Columbia Business School, Columbia University, New York, NY, United States; ^5^Digital Economy Lab, Stanford University, Stanford, CA, United States; ^6^Department of Biostatistics, Fielding School of Public Health, University of California, Los Angeles, Los Angeles, CA, United States; ^7^Fixed Income, Franklin Templeton Investments, New York, NY, United States

**Keywords:** behavioral economics, COVID-19, political polarization, information treatment, expectations, beliefs, media

## Abstract

We investigate the role of information exposure in shaping attitudes and behaviors related to the SARS-CoV-2 (COVID-19) pandemic and whether baseline political affiliation and news diet mediate effects. In December 2020, we randomly assigned 5,009 U.S. adults to nine brief text-based segments related to the dynamics of the pandemic and the safety of various behaviors, estimating the effects on 15 binary outcomes related to COVID-19 policy preferences, expected consumer behavior, and beliefs about safety. Average effects reach significance (95% CI) in 47 out of 120 models and equal 7.4 ppt. The baseline effects are large for all outcomes except beliefs. By contrast, interaction effects by political party and media diet are significant for beliefs but rarely significant for policy and behavioral attitudes. These findings suggest partisan policy and behavioral gaps are driven, at least in part, by exposure to different information and that equalizing information sources would lead to partisan convergence in beliefs.

## 1. Introduction

There is a large theoretical and empirical literature about the determinants of beliefs and expectations, together with their resulting effects on economic and social activity. However, recent evidence shows that political party affiliation is also an important explanatory variable for policy preferences (1), including those related to the COVID-19 pandemic (2–5). Given rising political polarization in the United States (6, 7), which is characterized by lack of inter-party cooperation among politicians and increasing dislike and distrust of partisan opponents, it is unlikely that a close link between partisan politics and public health beliefs will result in accurate beliefs or optimal policies (4, 8).

Misinformed beliefs and self-serving biases can have negative consequences for individuals, firms, and societies (9–13). If misinformation stems from exposure to heterogeneous information sources, then providing common sources of high-quality information should lower the incidence of erroneous beliefs and encourage more cooperative civic behavior across partisan lines, for example with income inequality (14, 15), inflation (16), innovation policy (17), and financial decision-making (18). Alternatively, if beliefs are tied to other dimensions identity, then information may either have no effect or be interpreted differently, depending on prior convictions and affiliations. In the context of a pandemic, clarifying these relationships is crucial to promoting scientific and cognitive literacy for optimal decision-making.

To understand the effects of exposure to COVID-19 related information on policy preferences, consumer behavior, and beliefs, we conduct a randomized controlled experiment using the Franklin Templeton-Gallup Economics of Recovery Study containing nationally representative data from 5,009 U.S. adults in early December 2020. Respondents were randomly assigned to one of nine information conditions described in Table 1 and their policy preferences, intentions to engage in public-facing consumption, and more general beliefs about the pandemic were subsequently collected across 15 survey items.

**Table 1:** Randomly assigned information segments.

1. The American Academy of Pediatrics—a leading group of physicians who guide health policy regarding children—has issued the following statement: “The AAP strongly advocates that all policy considerations for the coming school year should start with a goal of having students physically present in school. The importance of in-person learning is well-documented, and there is already evidence of the negative impacts on children because of school closures in the spring of 2020”.
2. As of October 31, 2020, the Centers for Disease Control (CDC) reports that there have been 192 deaths from the coronavirus for U.S. children aged 14 and younger, which accounts for 0.09% of all deaths from the virus.
3. According to data from the Centers for Disease Control (CDC), 2,715 Americans died per day from the coronavirus in the seven-day period ending April 22. Since then, the most deaths over a seven day period occurred on August 10, averaging 1,154 per day. The week ending November 10th saw 957 deaths per day.
4. November 13th saw a daily record for number of new confirmed coronavirus cases in the United States at 171,376.
5. Economist Emily Oster of Brown University has analyzed coronavirus data from a large number of schools and counties across the country. As of early October, the number of confirmed cases per day was 7 for every 100,000 students and 14 for every 100,000 teachers. These numbers are similar to rates in the neighboring county. Dr. Oster's analysis suggests that schools are not major sources of COVID-19 spread.
6. In Missouri, two coronavirus-infected hair stylists saw 139 clients. None of the clients developed symptoms, and of the 67 tested, all tested negative for the coronavirus. Both stylists and clients wore masks throughout the encounter. CDC officials concluded that masks prevented the spread of the virus.
7. In a flight from Italy to South Korea in April, 6 passengers unknowingly had the coronavirus, but they infected only one of 299 passengers–a woman who likely used a bathroom after an infected person, according to a report from South Korea's Centers for Disease Control. All passengers were wearing masks.
8. There are currently 77,000 Americans in the hospital with the coronavirus. That is higher than the previous peak of 60,000 hospitalizations in April.
9. According to the U.S. Bureau of Labor Statistics, the unemployment rate in October is 6.9%.

The text reports the wording of the segments that were randomly assigned to survey respondents.

We begin by documenting several new facts, including large gaps across political affiliation in policy preferences, consumer behavior, and information sources. For example, 72% of Republicans and 54% of Democrats support in-person elementary and secondary schooling, and Republicans are 21 percentage points (ppt) more likely to say they are likely to eat out within the next 30 days. We also find that Republicans and Independents consume a more politically diverse news diet than Democrats, consistent with literature showing the dominant influence of highly-professional, but politically left-leaning media (19).

Our primary research objective is to estimate the causal effect of providing information on eight policy preferences, four consumer behavior, and three beliefs. Our randomly-assigned segments vary in terms of emphasizing alarming news (e.g., record high cases) or more reassuring news (e.g. the low-probability of infection while wearing a mask on a flight). We test the causal effect of eight distinct treatments (each corresponding to a different information segment) on 15 outcomes. Each of the 15 models in our preferred baseline specification contains eight tests.

Results show large effects on policy and consumer-related attitudes following the introduction of this randomized information. Our reference group is informed that new daily COVID-19 cases have recently reached a record, which was true at the time of the survey. Compared to that group, providing people an alternative piece of information changes the likelihood of engaging in public- facing consumer behavior by 5.4 ppt in our preferred modeling structure. The point estimate is significant (95% CI) in 69% of tests, with a mean of 6.5 ppt when significant. The mean effect on policy preferences is 4.7 ppt, and those are significant in 36% of tests (8.3 ppt mean effect when significant). For example, informing people about the American Academy of Pediatrics (AAP) recommendation of having students learn in-person in the coming year is associated with a 15 ppt rise in the probability of supporting in-person elementary and secondary schooling. Similarly, it has an 11 ppt effect on the probability of eating out within the next month, and an 8 ppt decrease in supporting the closure of non-essential businesses.

Contrary to our expectations, we do not observe significant effects on our belief outcomes, which measure confidence in avoiding infection, the belief that COVID-19 is under control in the respondent's state, and plans to take the forthcoming COVID-19 vaccination. The mean effect is just 2.1 ppt and the results are significant in only 8% of tests. While our treatments did not directly address these issues, we predicted that reassuring information would boost confidence and alarming information would decrease feelings that the pandemic is under control.

With a few exceptions, we reject the hypothesis that these effects on consumer behavior and policy vary by political orientation or news diet. For consumer behavior, 0% of our tests for different effect for Democrats compared to Republicans are significant and 13% when we test a liberal-leaning diet against a right-leaning diet. For policy outcomes, only 16% are significant for partisan interaction effects and 23% for media diet interactions. On the other hand, we find heterogeneous effects of information on belief outcomes. 25% of tests reach significance when we examine interactions between party or news diet and beliefs. These results show consistent evidence that Democrats gain additional confidence and a greater sense of control relative to Republicans when exposed to our more reassuring news segments.

We interpret these findings as consistent with a theoretical framing that allows for attitudes to emerge based on the cumulative exposure to information sources. Partisanship and a partisan news diet provide filtering mechanisms that alter information exposure. New information alters attitudes. We find very weak evidence, however, that partisanship and prior news consumption distorts the interpretation of new information, at least in the context of a novel social phenomenon like the COVID-19 pandemic.

Our paper makes two primary contributions. The first is to identify precise measures of how much information affects self-reported policy-preferences and other attitudes collected immediately after exposure. More broadly, these causal estimates help clarify how cultural or partisan gaps in attitudes, behavior, and policy preferences may emerge as a result of information exposure. Our analysis of partisanship and the news diet distinguishes our paper from many information experiments in the literature, many of which are reviewed by Haaland et al. (20). In relevant work, Faia et al. (21) studies how partisan views interact with information selection and processing, leading media consumers to filter news that complements their prior convictions. The self-selection of information can gradually coalesce into biased but firmly-held attitudes, making exposure to countervailing information important in truth discovery. Coppock et al. (22) randomly assigned participants to read opinion pieces and found long-lasting effects on beliefs that were similar across political parties. Likewise, Nyhan and Reifler (23) find that information treatments reduce mistaken beliefs and that the effects do not strongly depend on prior attitudes. Haaland and Roth (24) and Abascal (25) found that information led to partisan convergence in beliefs but not policy positions on the topics of race and immigration, respectively. Whether new information has similar or heterogeneous effects on beliefs is crucial to theories dealing with the “polarization of reality” (1). We also provide relevant evidence on the pandemic, which was not initially polarized because it was a novel social context.

Secondly, our paper also builds on a number of recent contributions over the COVID-19 pandemic and provides important context to literature on optimal public health policy and the public's understanding of official recommendations and compliance with them. As has been well- established, many adults are misinformed about COVID-19 (26) and there are notable partisan gaps in attitudes, policies, and behaviors (2, 4). Atkesson et al. (27) finds that individuals dramatically overestimate the infectiousness and pathogenicity of COVID-19 and that providing people with expert analysis partially corrects their beliefs, with important mitigation-related behavioral implications. We validate these results and extend them to policy preferences and consumer behaviors. Likewise, in an experimental setting, Torres et al. (28) show that physician-delivered information increased COVID-19 knowledge, information-seeking, and self-reported protective behaviors.

Our paper also builds on important non-experimental work that documents how exposure to alarming vs. reassuring news segments affected social distancing behavior early in the pandemic (3). Understanding the role of information in explaining misinformed beliefs about COVID-19 is important for identifying paths forward for an economic and social recovery, particularly given evidence that altered beliefs have long term economic consequences (29, 30).

## 2. Data and experimental setup

### 2.1. Data description

From December 1 to December 7, 2020, we launched an experiment using the Franklin Templeton-Gallup Economics of Recovery Study containing nationally representative data from 5,009 U.S. adults. Respondents were randomly assigned to one of nine conditions described in Table 1. These were akin to news or information that respondents may have seen as media headlines, shared social media content, or summary messages from radio or television segments.

Sample weights were included in the summary statistics and modeling. They were calculated by Gallup to correct for nonresponse and construct a nationally representative population. Nonresponse adjustments were made by adjusting the sample to match the national demographics of gender, age, race/ethnicity, region, education level, marital status and employment status. Demographic weighting targets were based on the Census Bureau's 2018 data release of the American Community Survey and the Current Population Survey (February 2020).

Segments that conveyed more or less alarming information, while requiring the segment to be accurate, fact-based, and objectively stated. The goal of the experiment was not to precisely test actual media content, which can often be hyperbolic, but the effects of high-quality information that could be readily sourced and confirmed from credible public agencies or experts.

For example, some respondents view the statement that the “American Academy of Pediatrics (AAP) strongly advocates that all policy considerations for the coming school year should start with a goal of having students physically present in school. The importance of in-person learning is well-documented and there is evidence of the negative impacts on children because of school closures in the spring of 2020”. However, others view the statement “there are currently 77,000 Americans in the hospital with the coronavirus. That is higher than the previous peak of 60,000 hospitalizations in April”. We also treat some with economic data–which may raise the salience of respondent concerns about how COVID-policies have harmed the economy–without an explicit public health context: “according to the U.S. Bureau of Labor Statistics, the unemployment rate in October is 6.9%”.

**Figure 1:**
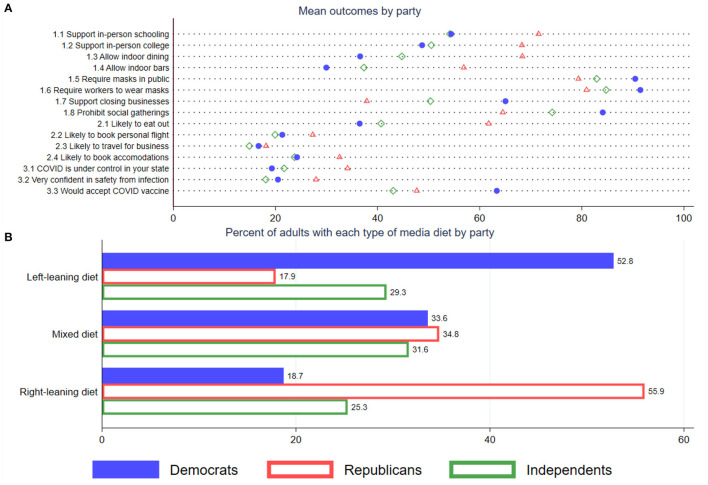
Heterogeneity in COVID-19 attitudes and news diet, by political affiliation. Data are from the Franklin-Templeton Gallup Economics of Recovery Study, fielded between December 1-7, 2020 with 5,009 U.S. adults responding. **(A)** reports the sample-weighted summary response to 15 outcomes coded on a binary scale by respondent political orientation. Numeric label distinguishes between (1) policies, (2) consumer behavior, and (3) beliefs. Independents include those who favor no party or favor a third party. Responses are weighted to be nationally representative. **(B)** reports the shares of Republicans, Independents, and Democrats who consume left-leaning, right-leaning, and mixed media diets based on the sources identified in their survey responses. See Supplementary material for further classification details.

Following our experiment, our survey collected data on 15 outcomes. This survey structure effectively constrained our analysis, since items collected before the experiment could not have been affected by the experiment. To ensure that we are fully comprehensive and not cherry- picking results, we report coefficients from all 15 items. We group them into three categories: (1) eight policy preferences (2) four consumer behavior (3) and three beliefs (see Figure 1). Policy preferences are captured as support or lack thereof for policies that would require mask use in public, place restrictions on in-person schooling, social gatherings, restaurants, and bars. Consumer behaviors related to plans within the next 30 days to book a reservation at a restaurant, a flight, or hotel accommodations. Beliefs capture general level of confidence in avoiding COVID-19 infection while out in public, whether the virus is under control in the respondent's state, and willingness to get the soon-to-be-released COVID-19 vaccine. The latter is, admittedly, not easily categorized with the others, as it involves public-health behavior, informed by beliefs about the vaccination process, efficacy, and safety. Analytically, it is convenient to group those three because our treatments do not provide direct information about vaccines or the other two “belief“ items, so our expectation is that our treatments will have a weaker effect on those outcomes.

Nine of our 15 outcome variables were collected as a binary response, and we coded the other six accordingly. For consumer behavior, responses are coded as 1 (for likely) if the respondent indicated either of the top two choices (somewhat likely or highly likely) and 0 otherwise. Respondents were coded as 1 if they were “very confident” they could protect themselves from COVID-19 infection while out in public on a 1–4 scale and 0 otherwise. Those who would agree to be vaccinated with the forthcoming COVID vaccine were coded as a 1, while those who replied “no” or “I don't know” were coded as 0. Supplementary material S1, S2 in the supplement present descriptive statistics on the incidence of each of these information treatments across the different outcome variables, which provides guidance for interpreting the marginal effects in Section 2.

### 2.2. Randomization

While randomization alleviates concerns of omitted variables bias, it does not ensure orthogonality between error terms and treatment assignment, as imbalance between groups can arise randomly. For each respondent, we observe a wide array of characteristics, including: binary variables for race and ethnicity, age, age-squared, and a cubic value, a binary variable for male sex, binary variables for levels of educational attainment, whether the respondent s a homeowner, political party affiliation, current employment status, household income (11 bins of fixed effects), political affiliation, pre-COVID travel behavior, pre-COVID indebtedness vs. saving, and self-reported medical risk factors for oneself and family members. We also include the results of a two-item numeracy test, because numeracy may be related to the ability to correctly interpret scientific guidelines and translate risk probabilities into appropriate behavior and attitudes. The supplement text provides details about how these data were collected and coded.

To test of whether lack of balance may bias the interpretation of mean differences on our outcomes by experimental group, we regressed assignment status for each of our nine experimental groups on a vector of the explanatory variables listed above and calculated the predicted values. The predicted values capture the probability that an individual with a given set of characteristics will be assigned randomly to one of the nine groups. Each respondent has nine such values. In a second set of regressions, we regress each of our 15 outcomes on demographically-predicted assignment. Significant values would suggest bias in assignment. The results are reassuring that our randomization strategy mitigated bias with respect to our outcomes of interest. Since there are nine experimental groups and 15 outcomes, there are 135 coefficients to test. Of these 135, only four (3%) were significant at 95% confidence intervals. In other words, random chance generated clusters of people with demographic characteristics that make them slightly more inclined toward an outcome of interest in only 3% of instances. These results are available upon request.

Since we could not completely rule out lack of balance in our randomization, our preferred models include our full list of demographic controls. We also attempt to rule out other sources of bias, such as local exposure risk, from unusually high or low deaths per capita or population density, using country level data. Supplementary Figure 1 shows how our preferred model performs against two alternative models: one with zero controls and one with additional controls for country-level risk. The results are broadly similar, though our baseline model enhances levels of significance for the consumer behaviors in some cases, largely by controlling for pre-COVID travel patterns that are highly correlated with consumer behavior during the pandemic.

Our experiment was conducted near the end of the survey, constraining possible outcome variables to those we intended to test. To promote replicability, our analysis includes all possible outcomes, and we show results with and without our list of extensive demographic controls (see Supplementary Figure S1 for meta-analysis comparison and Supplementary Figure S3 for results with no controls). All data and code are publicly available.

### 2.3. Descriptive statistics

Figure 1A documents substantial heterogeneity in policy preferences, beliefs, and consumer behavior by political affiliation. In particular, the mean absolute value gap between Democrats and Republicans is 16 ppt and ranges from 1 to 32. Gaps between Independents and Democrats are smaller (6 ppt) than gaps between Independents and Republicans (12 ppt). The starkest contrasts are over policy preferences: 37% of Democrats and 68% of Republicans support allowing indoor dining, whereas 65% of Democrats and 38% of Republicans support temporarily closing non-essential local businesses.

We also see large differences in support for in-person schooling and the likelihood of eating out. Interestingly, other consumer behavior, such as the likelihood of booking hotel accommodations or a flight personal travel are not as far apart (8 and 6 ppt gaps), while the likelihood of booking a flight for business travel is essentially the same (1 ppt difference).

One explanation behind the heterogeneity in policy preferences is a difference in the underlying tastes across individuals. Another is exposure to different information. Because of the way that we have designed our information intervention, we are able to rule out heterogeneity in preferences and focus on the contribution of information. To provide some motivation on the exposure to different information, Figure 1B examines the composition of individuals' media diet by exploring the type of news outlets that they pay attention to (see Supplementary material for an explanation of how we categorize news sources by political orientation based on the political views of its consumers, not the content provided).

As expected, most Republicans consume a news diet from sources that are largely consumed by other Republicans and vice versa for Democrats. For example, 53% of Democrats consumer a liberal-leaning diet, and 56% of Republicans consumer a right-leaning diet. Independents fall in-between. Importantly, less than one out of five Republicans and Democrats consume a news diet that consists of sources commonly viewed by the opposite party. Still, a mixed diet is common for roughly one third of our sample. This is consistent with empirical research showing that many Republicans consume center-left news sources (19).

## 3. Identification strategy

We exploit the random assignment to experimental conditions to estimate the causal effect of information on these policy preferences and consumer behaviors through the following:


$$y_{i}=\gamma INF\;O_{i}+\psi POL_{i}+\xi \left(INF\;O_{i}\times POL_{i}\right)+\beta X_{i}+\varepsilon_{i}$$

where y denotes our outcome of interest for person *i*, *INFO* denotes our information treatment, *POL* denotes a vector for political affiliation (Republican or Democrat, normalized to moderates), and *X* denotes our vector of individual demographic characteristics, and ε is our error.

While random assignment reduces the correlation between the error term and our demographic variables (in *X*), random imbalances between groups can emerge, as discussed above; thus, we include the *X* terms to guard against this potential bias. We focus on the γ coefficients since these represent the effects of information. In an extended model, we also analyze interaction effects between political affiliation and the treatment, which are captured through ξ. These effects are omitted in our baseline. Significant effects on the interaction term would suggest that treatment effects are heterogeneous across party affiliation, which could lead to belief convergence if the sign is the opposite of the effects captured for the same party through ψ. We also test models that replace the Democratic Party term and its interaction with whether or not the respondent is coded as having a left-leaning media diet. The results are similar to using partisanship as the interaction and are available in the Supplement (Supplementary material S5, Supplementary Figure S2).

Our experimental design does not have a natural control group. We could have omitted the news segment item for a random sub-group of respondents. The downside would be that we would have lost the opportunity to test another segment, and we still would have been left with a group of respondents who were exposed to COVID information outside the survey. We chose a segment to serve as our reference group that most closely approximated predominate national news coverage about COVID-19 in the United States.

To determine this, we used a tool created by Harvard University and affiliated partners called Media Cloud, which allows scholars and users to study the salience of words and phrases that appeared on news websites. We narrow our treatments to short searchable phrases to correspond to actual news stories that convey the same or a similar message. In the Supplement (Supplementary material S6), we show the results of our search. Treatment 4 is by far the most salient. The segment reads: “November 13th saw a daily record for number of new confirmed coronavirus cases in the United States at 171,376”. Our search required the phrase “COVID cases” and adjectives “peak” “high” “rising” or “soaring”. This appears in roughly half a million web-links, consisting of over one quarter of all websites during peak coverage. The segment was very close to actual headlines published around the middle of November in popular center-left sources, including: The ABC News (31), The Washington Post (32), and The New York Times (33). Yet, conservative media listeners consumed a very different analysis. Transcripts from Fox News hosts Tucker Carlson (34) and Greg Gutfeld (35) emphasized the undesirability of state-mandated COVID-19 restrictions and suggested the disease burden of COVID-19 had been exaggerated by Democrats. Using treatment 4 also allows for a convenient interpretation. Every other segment presents more reassuring news about COVID-19, so the effects reported below test whether more reassuring news affects our outcomes of interest. The supplement (Supplementary material S1) reports the results for all segments, so readers can easily calculate other effects.

Given the large number of hypotheses tested, we consider the possibility that random chance could generate significant results for some subset of our models. Accordingly, we check our preferred results against more conservative *p*-value thresholds, using an algorithm developed in the methodological literature, as well as more conventional corrections (36). We report these result in the supplement (Supplementary material S7). Even with these adjustments, we find that many of our tests remain significant.

We also consider the external validity of our survey results by focusing on the planned consumer behavior, which is readily aligned with timely objective data from external sources. For most U.S. states and the District of Columbia, the restaurant reservation platform OpenTable reports the number of people who dined in restaurants in December 2020 compared to the same days in February 2019. We aggregated all of December and correlated the values with the percentage of people living in the same state who reported in the December Franklin-Templeton Gallup Economics of Recovery Study that they were likely to eat at a restaurant that month. The correlation was moderately high (0.35) and became high (0.51) when excluded states that had fewer than 50 respondents (13 states out of 41). We replicated this exercise with a slightly larger group of states (49 and 28) using the Google Community Mobility index—which is based on the geo-tracking of cell phones—and restricting the data to December 2020 visits to restaurants, cafes, and related shopping experiences. Since Google releases county-level data, we matched these data to our respondents at the county level, which is an advantage over the OpenTable analysis. The correlations between Google mobility visits with self-reported dining plans were even higher (0.40 and 0.65). These results are shown in Supplementary Figure S4 in the supplement. Taken together, this exercise demonstrates that our survey item on plans for eating out for the month ahead corresponded to actual behavior over the subsequent month. It follows that our treatment effects are likely to have real-world implications.

## 4. Main results

**Figure 2:**
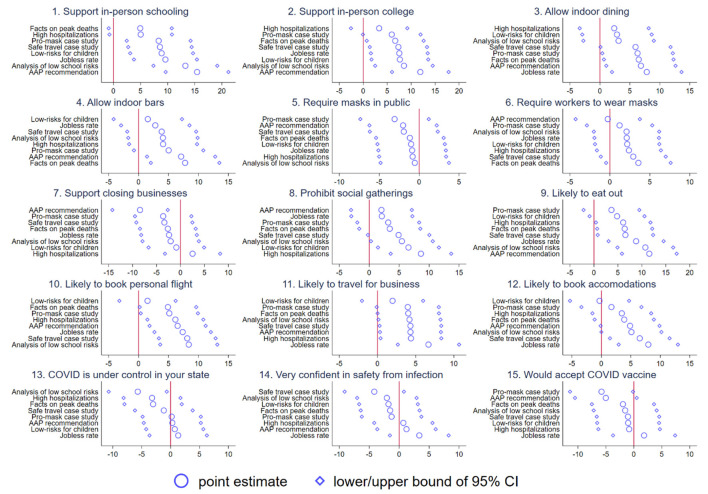
The causal effect of COVID-19 information on policy preferences, consumer behavior, and beliefs. Each figure plots the coefficients from the baseline model. 95% confidence intervals are shown.

We estimate Equation 1 for 15 outcome variables and each of the eight information interventions, relative to the omitted group of record high cases. Starting with Figure 2, we find that there are large causal effects of information on most of our policy outcomes and consumer behaviors. For example, individuals who receive the AAP recommendation are nearly 15pp more likely to support in-person schooling, 13 pp more likely to support in-person college, 7 pp more likely to support indoor dining, and 10 pp more likely to support indoor bars (significant at the 5% level) 0.5 We find similarly large and intuitive effects for many of our other information treatments. For example, providing information on the low risk of transmission for children or analysis on the low risk of school reopenings is associated with a 10–15 pp rise in the probability of supporting in-person schooling and in-person college. However, the effects on support for indoor dining are somewhat smaller. The varying treatment effects are a validation test: if respondents were simply reacting to some omitted factor, they would react to the information interventions similarly. Turning toward consumer behavior, we see fairly meaningful effects of the information on the AAP recommendation, analysis of low school risks, jobless rate, safe travel case study, and facts on peak death on the probability of eating out, booking a personal flight, traveling for business, and booking accommodations, averaging roughly 5–10 ppt. These effects are intuitive. For example, information on the jobless rate puts coronavirus cases in context of the economic reality that the country was in as of December 2020. Further, the AAP recommendation, safe travel case study, and analysis of low school transmission risk all help mitigate fear about coronavirus by highlighting that it is not as easy to catch as the media often made it out to be. Finally, information on peak deaths puts the numbers in context of the crisis in April 2020.

Finally, we turn toward policy preferences and beliefs about the pandemic. In general, we find limited associations with mask requirements in public and requirements for workers to wear masks. While people generally become more supportive of requiring workers to wear masks, they become less supportive of mask requirements in public. In both cases, however, we fail to reject the null of no effect. Similarly, we find null effects on whether respondents believe that the coronavirus is under control in their state, whether they are safe from infection, and whether they would accept the vaccine. Both the pro masking case study and AAP recommendation make people roughly 5 ppt less likely to accept a hypothetical vaccine.

**Figure 3:**
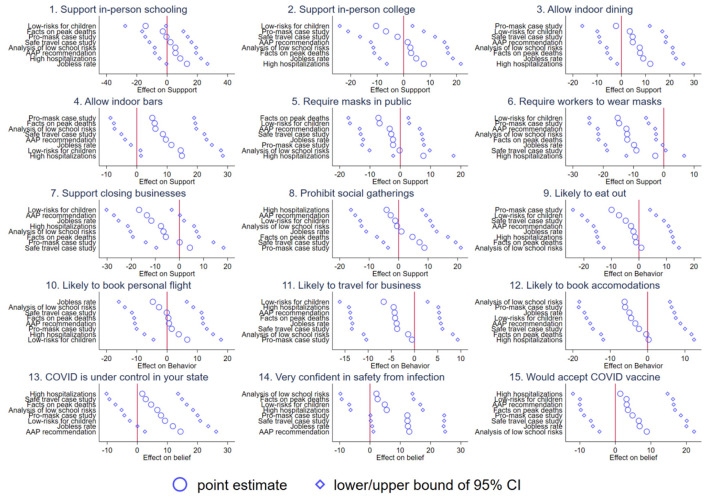
Treatment effects of democratic party members relative to Republican Party Members. Each figure plots treatment effects for respondents identifying as Democrats relative to Republicans. 95% confidence intervals are shown.

Next, Figure 3 potential heterogeneity by political affiliation. For ease of interpretation, we report the interaction effect between an indicator for identifying as Democrat, normalized to identifying as Republican; we drop Independents. Importantly, these coefficients are not the mean effects—they are the interaction effects, i.e., ξ from Equation 1. In general, in nearly all cases, we fail to reject the null that there is no treatment effect of the information on policy preferences, consumer behavior, or beliefs about coronavirus. That is important since it suggests the potential for convergence—that is, a similar response among Democrats and Republicans when presented with information in a controlled and less polarized setting. While some of the point estimates are economically meaningful, the confidence intervals are usually large enough to include zero.

**Figure 4:**
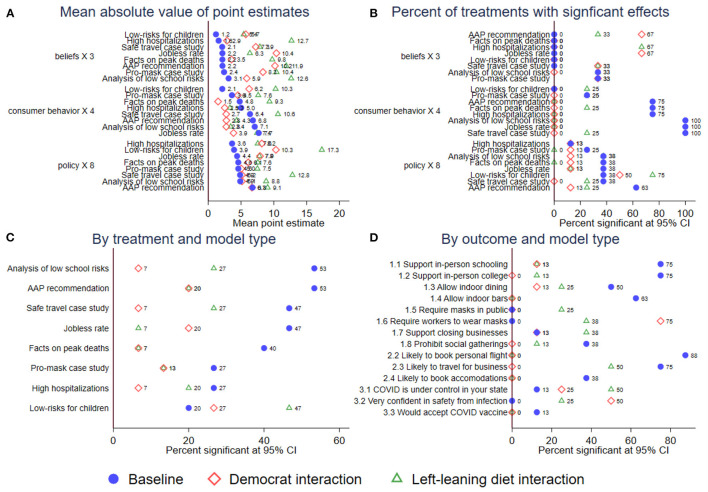
Meta-analysis of treatment effects across model types. Each figure plots meta-data from our baseline model with the full set of controls and no interactions, a model that includes an interaction term for Democrats relative to Republicans, and a model that includes an interaction term for consuming a liberal-leaning diet relative to a right-leaning diet. **(A)** shows the mean point estimate for each experimental group organized by the three outcome types. **(B)** show the percentage of treatment effects reaching significance thresholds with 95% confidence intervals for each experimental group organized by the three outcome types. **(C)** shows the percentage of treatment effects reaching significance thresholds with 95% confidence intervals for each experimental group, aggregated across all treatment. **(D)** shows the percentage of treatment effects reaching significance thresholds with 95% confidence intervals for each experimental group, aggregated across all outcomes.

To facilitate the interpretation of all these results, Figure 4 summarizes the results from a meta-analysis that presents the coefficients obtained from 120 treatment effects (eight treatments for 15 outcomes) in our baseline model and in two interaction models for a total of 360 coefficients. Panel A provides the point estimates across all 360 tests and Panel B reports the percentage of these tests that reach significance thresholds. Furthermore, Panel C aggregates the percent significant by treatment and Panel D aggregates the percent treatment by outcome. In this sense, Figure 4 allows readers to see which treatments were the most consistently effective. Treatments that included re-assuring expert analysis and not just facts had the largest effects, though facts were also significant in many cases. As for outcomes, expected consumer behavior was the most consistently affected by our treatments, suggesting that COVID-19 related information has important real-world implications on the economy and on social contact.

Consistent with our earlier results in Figure 3, the meta-analysis shows that most of our interaction effects were not significant with the exception of those pertaining to two of our “belief” items: general confidence in avoiding infection and the assertion that COVID-19 is under control in the respondent's state. Democrats and those with a left-leaning news diet displayed significantly larger effects than Republicans or those with a right-leaning news diet.

## 5. Discussion

### 5.1. The value of information

There is an active debate, perhaps now more than ever, about the causes and remedies to di- vergence in beliefs, especially in an era with such a large quantity of content proliferating the web and social media. While there is a general understanding that heterogeneity in individual preferences—that is, time-invariant characteristics and tastes—are important determinants of policy preferences and consumer behavior, there is much less agreement on the causal effect of information, particularly over the coronavirus. Our paper reports the results from a novel field experiment conducted within the first year of the COVID-19 pandemic to answer these questions. We find that short fact-based information segments about COVID-19 have large causal effects on policy preferences and consumer behavior. These effects are largely consistent across political parties and media diets. When it comes to beliefs about the safety of being in public and the extent to which the pandemic is under control locally, respondents were largely unaffected by our information segments, but Democrats and those with a left-leaning media diet showed a stronger response than Republicans and those with a right-leaning diet, resulting in belief convergence.

High-quality information is costly to obtain and interpret correctly. In the Supplementary material, we provide a simple theoretical discussion of a model where consumers drift apart and remain on islands of partial knowledge in part because the price and quality of information is hard to discover and evaluate. Our model is consistent with this theoretical framing and suggests that repeated exposure to different information could explain the observed patterns in attitudes. Given the different media environments documented here (and the literature discussed above), we assume that Democrats and Republicans have been exposed to different information relevant to COVID-19. However, the effects of information seem to be largely independent across groups with heterogeneous baseline beliefs, but the effects are not enough to eliminate differences between partisan groups (see Figures 3, Supplementary material S2). That suggests eliminating partisan polarization in beliefs and behaviors would take long-term exposure to the same sources of information, though we cannot provide direct evidence for this in our short-term experiment.

Our rejection of interaction effects (between partisanship or news diet and our experimental treatments) is consistent with previous work that partisans update their beliefs when presented with relevant facts and that psychologically affirmative statements have no additional effects (23). In this case, partisans update beliefs even when it goes against the norms of their fellow partisans. One explanation, and potential contrast with other information experiments on viruses (37), stems from the way our intervention was implemented: we provided succinct facts largely separated from some of the politicization that has been taking place over the pandemic (4). Such politicization, especially in the presence of network effects (38), can heavily affect beliefs.

It is also possible that salience and attention matter more than the cumulative effects of prior exposure. If so, our respondents may briefly update their views because of the information we put in front of them without deeply considering the implications or applying them going forward. A limitation of our research is that we do not follow participants over time to see how long these effects last. Yet, using similar techniques, Coppock et al. (22) finds evidence of long-lasting effects from the random assignment of longer texts. Another limitation is that we do not directly reserve consumer behavior, only self-reported intentions to engage in various behaviors. Randomly assigning people to different media diets over a long period of time and observing real-world behavior–such as restaurant outings–in addition to policy preferences and beliefs would be a more compelling and comprehensive test of the power of information, albeit difficult to implement.

### 5.2. Context and limitations

Given the massive economic and social harm inflicted by the COVID-19 pandemic and resulting policies, our results underscore that access to high-quality information is likely to have important economic and social effects at scale. In fact, such information not only influences the policies that are likely to get adopted (e.g., severity and duration), but also the degree of fear, and therefore, consumer behavior that follows, impacting economic activity. On one hand, exaggerating the risk will lead to too little economic activity to sustain the standard quality of life, as well as the additional effects on mental health and wellbeing. On the other hand, understating the risk could lead to insufficient mitigation behavior that accelerates the transmission of the virus.

Our research nonetheless contains several limitations. First, we only fielded a single study that is a snapshot of a moment in time. It is possible, for example, that the value of information has evolved over time and that it is less useful in even more politicized environments. We would like to see future work that tracks the behaviors and beliefs of respondents over time.

Second, while we asked about a respondent's media diet, we would like to explore the effects of information in more natural settings and how the source of the information influences beliefs. Especially in an era of social media, we would like to test how intensity of social media use and source exposure influences beliefs and behavior. Third, we would like to understand more about the external validity of these results in other contexts beyond the coronavirus and in other cultural and geographic contexts outside the United States. We leave these questions for future research.

Given the severity and duration of the COVID-19 pandemic and associated government restrictions, a large question remains over the effects of enduring beliefs on economic activity and how best to understand and correct misinformed beliefs.

## 6. Conclusion

Our results provide, to our knowledge, the first causal evidence on the effects of information on both policy preferences and intended consumer behavior. The results are particularly novel and meaningful given that they involve pandemic related policies–which are highly contentious and salient–and consumer behavior relevant to activities that have seen depressed revenue in the United States and globally because of disease suppression efforts. In this sense, our results help clarify how public debate and media coverage about COVID-19 has contributed to policy preferences and consumer behavior. Additionally, we study the role of partisanship and news diet in potentially generating heterogeneous treatment effects and conclude that, for the most part, adults respond similarly to new high-quality information, despite partisan-based disparity in baseline beliefs. The exception to this finding is that reassuring information brings Democrats closer to Republicans in terms of how they understand their personal risk and whether the pandemic is under control locally.

Nonetheless, our paper leaves several areas for further research. First, do individuals who update their beliefs in response to high-quality information maintain the more accurate beliefs, or does accuracy degrade over time and at what speed? A related question is whether information treatments affect the self-selection of subsequent information. Understanding how to empower consumers to seek out high-quality information is an important implication of this work, and on the supply side, it is also important to better understand how high-quality information can be produced and distributed, especially as it relates to public health information during a pandemic. A secondary series of issues arises as to how more informed individuals might interact with and potentially persuade others on social media and in-person. Finally, at an aggregate level, how long does it take for beliefs to converge or diverge under different plausible scenarios?

## Data availability statement

The raw data supporting the conclusions of this article will be made available by the authors, without undue reservation.

## Ethics statement

Our study was reviewed by Gallup's Internal Review Board, which is registered with the U.S. Office of Human Resource Protection with number IRB00004888. Written informed consent for participation was not required for this study in accordance with the national legislation and the institutional requirements.

## Author contributions

Conceptualization: JR and SD. Methodology: JR and CM. Investigation and visualization: JR. Supervision and writing—original draft: JR, CM, CR, and SD. Writing—review and editing: JR, CM, and CR. All authors contributed to the article and approved the submitted version.
