# Supplementary material for: Information, partisanship, and preferences in a pandemic
Source: Front Public Health. 2023 Mar 8;11:1019206. doi: 10.3389/fpubh.2023.1019206 (PMC10031094; doi:10.3389/fpubh.2023.1019206)
Supplement: Supplementary file 2 [file Data_Sheet_2.docx]

Supplementary Materials for

**Information, Partisanship and Preferences in a Pandemic**

Jonathan Rothwell*, Christos Makridis, Christina Ramirez, Sonal Desai

*Corresponding author. Email: [Jonathan_Rothwell@Gallup.com](mailto:Jonathan_Rothwell@Gallup.com)

**This PDF file includes:**

Supplementary Text

Figs. S1 to S4

References 1 to 2

**Other Supplementary Materials for this manuscript include the following:**

Data S1 to S8

Supplementary Text

Theory: how information heterogeneity can persist and lead to polarized beliefs

There has been a long-held recognition that information as costly to acquire, requiring search and evaluation Stigler (1961). Developing a trusted source for information lowers those costs, but only results in someone being more informed if the trusted source is truly high-quality. We begin with the recognition that the value of information is a function of quality and price. However, unlike other goods, the quality of information is very difficult to know prior to consuming it and is even difficult to know retrospectively. These two facets of information create unique and significant difficulties for consumers. Trust becomes especially important, but trust is disconnected from objective verification, allowing for the formation of stable and heterogeneous beliefs.

Our theory predicts that providing consumers free high-quality information from a trusted source results in the convergence of beliefs. Our presupposition is that consumers generally lack access to low-cost high-quality information. Suppose that the value of information is equal to the quality divided by the price.

We assume that the price of information is increasing with its value because relevant, non-obvious information is costly to discover and disseminate; information is also costly to interpret correctly because the sound interpretation of facts often requires expertise. Traditionally, these costs have been funded by some combination of advertisement fees and subscription fees, and desire for fame or influence persuades many experts or quasi-experts to donate interpretations to the media. Non-media sources of information also face costs, such as government agencies and privately funded surveys, and communication within and across social networks, which are usually unknown to the media and public. The internet and social media platforms, funded via advertisements, have dramatically lowered the price of access to many types of information, including the political views of influential people and those in one’s social network.

The price of information is difficult to determine, consisting of partial but vague signals. Platforms that offer news may be free to the consumer but rest on cheap or expensive data gathering processes that are unknown to the consumer. Moreover, consumers do not know, in most cases, how costly it would be for them to acquire the knowledge to answer their questions or discover important information or interpret it correctly.

Information quality is also mostly unknown prior to consumption, and unlike many tradable goods, like automobiles, there are no well-defined specifications. Further, unlike many services, such as movies and restaurants, customer reviews and ratings are not available. The probability that a piece of information will be of the highest quality (e.g. a relevant, insightful, and true fact) has to be estimated and varies from 0 to 1. An individual’s degree of knowledge about public facts (informativeness) is the aggregate value of all consumed information sources multiplied by the probability that the information has perfect quality.

To summarize, whenever distinct sources of information prevail, informativeness varies because price, quality, and value are all difficult to determine, creating large inefficiencies in the market for information. Many consumers would pay more for higher quality news, but cognitive biases (e.g., party and news source biases) can shape many of the consumption patterns by altering the information that they pay attention to and process.

The news industry attempts to solve these problems by imposing editorial standards and professional codes of conduct, but participants are not forced to adhere to these standards and are often rewarded financially for violating them.

If beliefs are a function of exposure to information, it follows that beliefs will become segmented by information sources and vary based on the quality of those sources. These dynamics create islands of beliefs, with varying correspondence to reality.

Survey details

We launched a novel survey and randomized experiment through a partnership between Gallup and Franklin Templeton, called the Economics of Recovery Study, which took place in six waves from July to December 2020. We use data from wave 6 of the survey, which was fielded between December 1 and December 7th, 2020. Results from this study are based on self-administered web surveys from an opt-in sample provided by Dynata. Gallup weighted the obtained sample to correct for nonresponse.

We make nonresponse adjustments by adjusting the sample to match the national demographics of gender, age, race/ethnicity, region, level of education, marital status, and employment status. Demographic weighting targets are based on the Census Bureau’s 2018 data release of the American Community Survey and the Current Population Survey (February 2020).

Our list of control variables included a measure of numeracy, adapted from the Berlin Numeracy Test (Cokely 2012). Our item is based on the following two survey questions:

Please answer the questions below to the best of your ability. Do not use a calculator, but feel free to use paper and pencil.

Q1 Imagine you are throwing one standard (six-sided) die 6 times.

How many times would you expect this die to show the number 1 or the number 6?

Q2 Imagine that exactly half of people who are infected with a new virus show symptoms. The other half show no symptoms. Now imagine a town of 1,000 people where 200 people are showing symptoms.

How many people in the town would you estimate are infected?

60% of respondents scored “0”, meaning that they got neither right. 26% got one right, and 14% got both right. There was no correlation between this item and political party or news diet, but it was correlated with having a graduate degree (r=.13).

Classification of News Diet

Our survey also collects data on media sources. To avoid making the respondent consider every media source, we start by asking how many hours per week respondents devote to news consumption. 95% responded with a value above zero and were then asked what form of media “do you get most of your news?” Respondents can choose television, radio or podcasts, newspapers, online, or social media. Then, the respondent is presented with a list of 10-15 prominent news sources for whichever form (e.g. *New York Times*, *Wall Street Journal* for print; Fox and CNN for television, etc), and asked to select each one they “normally” consume (i.e. read, watch, or listen to). To code these on a left-right spectrum, we calculate the Pearson correlation coefficient (using survey weights) for an underlying respondent-level indicator of political orientation, using a combination of both their 2020 vote and their stated party affiliation.

Each of the two political variables (vote and party) was coded on a -1,0,1 scale, with 0 indicating neutrality (encompassing non-voters, third-party voters or members, and independents). This political index is correlated with consumption of each media source. The results conform with our subjective impressions of the political orientation of sources and information from other sources. For example, Fox News is strongly oriented towards the right with a value of 0.25; MSNBC and CNN has values of -0.25 and -0.33, respectively; Rush Limbaugh and Sean Hannity radio programs have values of 0.29 and 0.27, respectively; All Things Considered (an NPR program preferred by Democrats) has a value of -0.27. The Wall Street Journal has a value of 0.06, indicating a weak correlation with right-leaning readers, consistent with its centrist orientation. The correlation coefficient is then averaged across individuals to create a mean diet index, which is coded as left, mixed, or right, depending on whether values were <=-0.1, between -0.1 and 0.1, or >=0.1.

Fig. S1.

Meta analysis of baseline model with all respondents with various assumptions about the optimal set of controls. Top panel shows the mean absolute point estimates of experimental effects on outcomes from a model with no control variables (solid blue dot), our preferred model with demographic controls for all adults (red triangle), and an additional model with all of the demographic controls plus controls for local COVID-19 risk (the log of cumulative COVID-19 deaths per capita in the respondent’s county through November 2020 from USA Facts and county population density from the Census Bureau and American Community Survey). The bottom panel shows the percentage of coefficients from these models that reach significance at 95% confidence intervals. Outcomes are categorized as beliefs, consumer behavior, and policy.


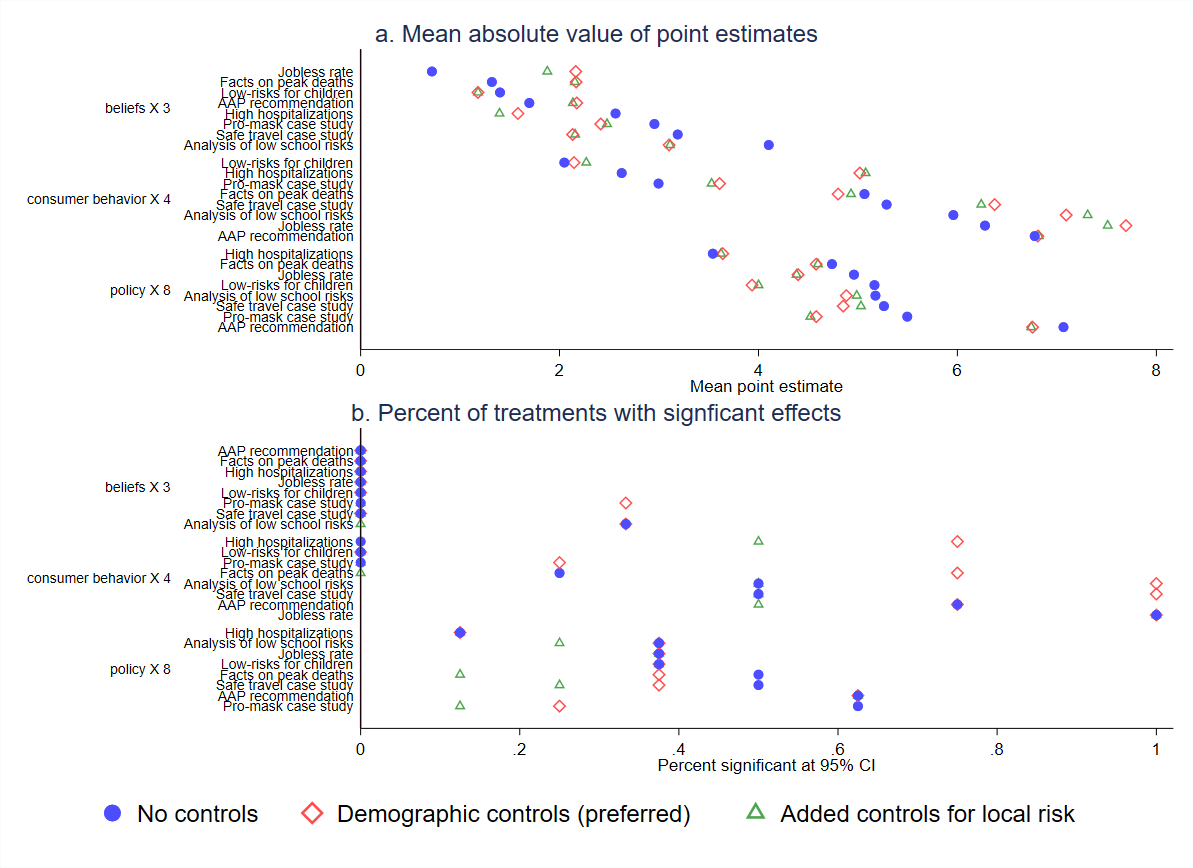


Fig. S2.

Treatment effects for those with a Left-leaning Diet Relative to those with a Right-leaning Diet. Each figure plots treatment effects for respondents identified as consuming a left-leaning news diet relative to a right-leaning diet. Effects are identified in a model that excludes those with a mixed-media diet and is in relation to the experimental reference group. Models 1-8 are policy preferences. Models 9-12 are consumer behaviors, and 13-15 are beliefs. All models control for the demographic characteristics listed in text, including: age, education, race, employment status, political affiliation, numeracy, medical risk factors, household income, health status, and gender. 95% confidence intervals are shown.


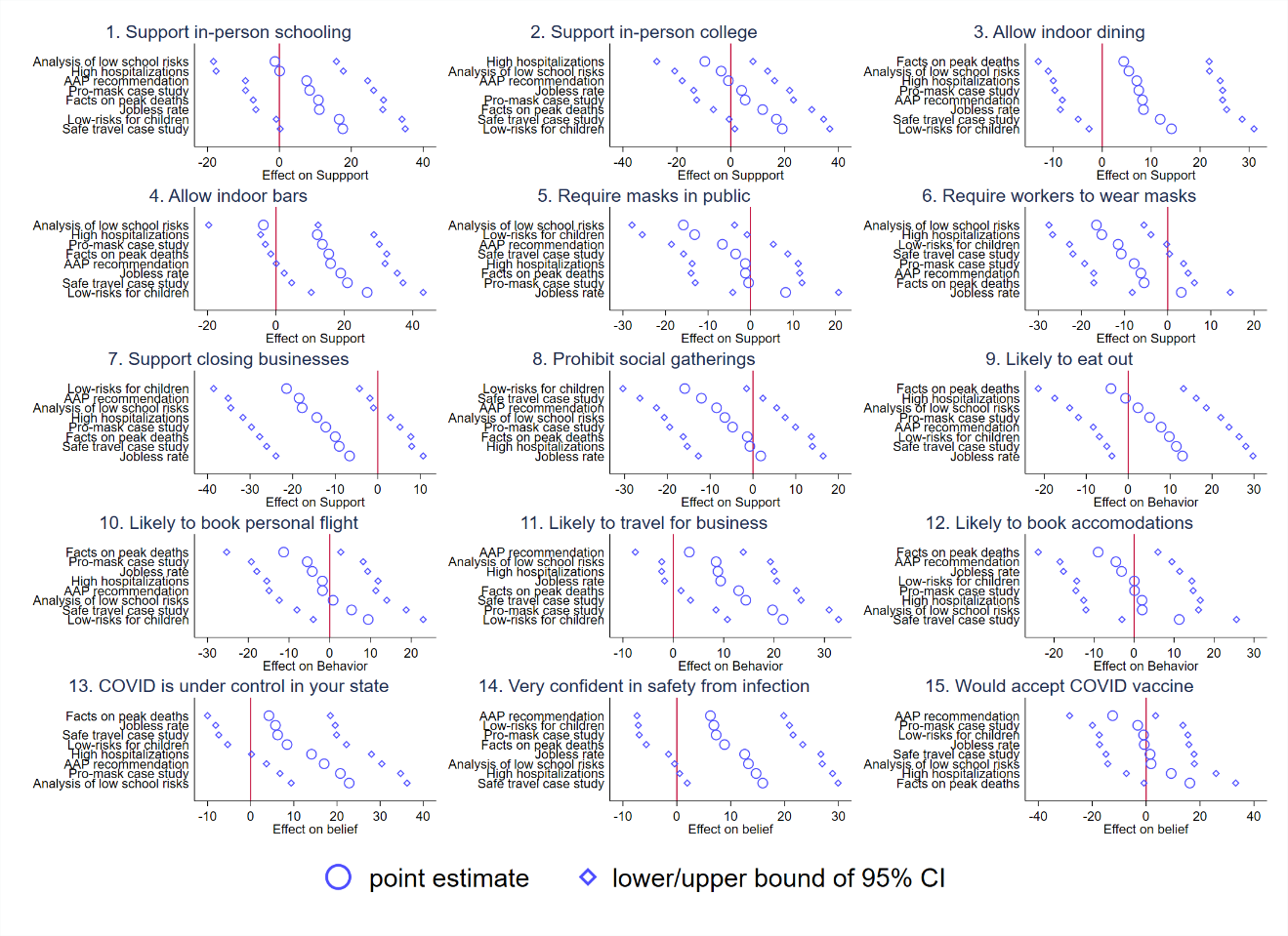


Fig. S3.

Treatment effects in baseline model with no adjustments. Each figure plots the coefficients from the baseline model associated with 8 treatments relative to the reference group. Models 1-8 are policy preferences. Models 9-12 are consumer behaviors, and 13-15 are beliefs. No control variables are included. 95% confidence intervals are shown.


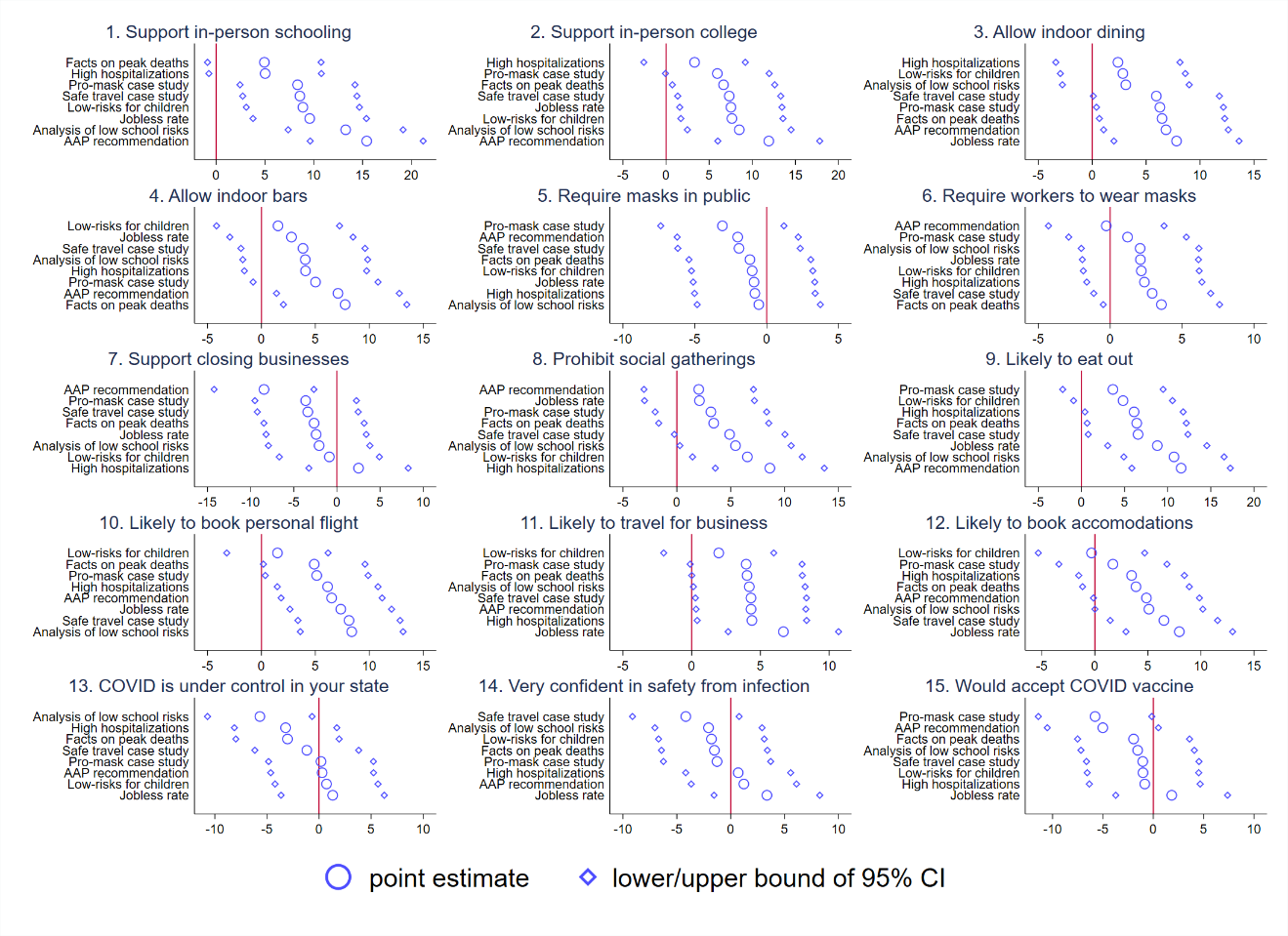


Fig. S4.

Self-reported resturant plans and actual consumer behaivor at the state level. This figure plots objective measures of consumer behavior and the self-reported percent of respondents who report they are likely to eat at a restaurant in the next month by state for the period of our survey, December 1 to December 7, 2020. Plots 1 and 2 show an index from Open Table that compares actual resturant diners in December 2020 to February 2020, with values above one showing growth in dining relative to the pre-pandemic baseline. Plots 3 and 4 replace the OpenTable index with December Google Mobiltiy for “retail,” which, according to Google, refers to mobility trends for places like restaurants, cafes, shopping centers, theme parks, museums, libraries, and movie theaters and is also indexed to before the pandemic. Plots 2 and 4 restrict to states with at least 50 respondents in the underlying sample. The Pearson correlation coefficients are 0.35, 0.51, 0.40, and 0.65, respectively, across the four plots.^[[1]](#footnote-1)^


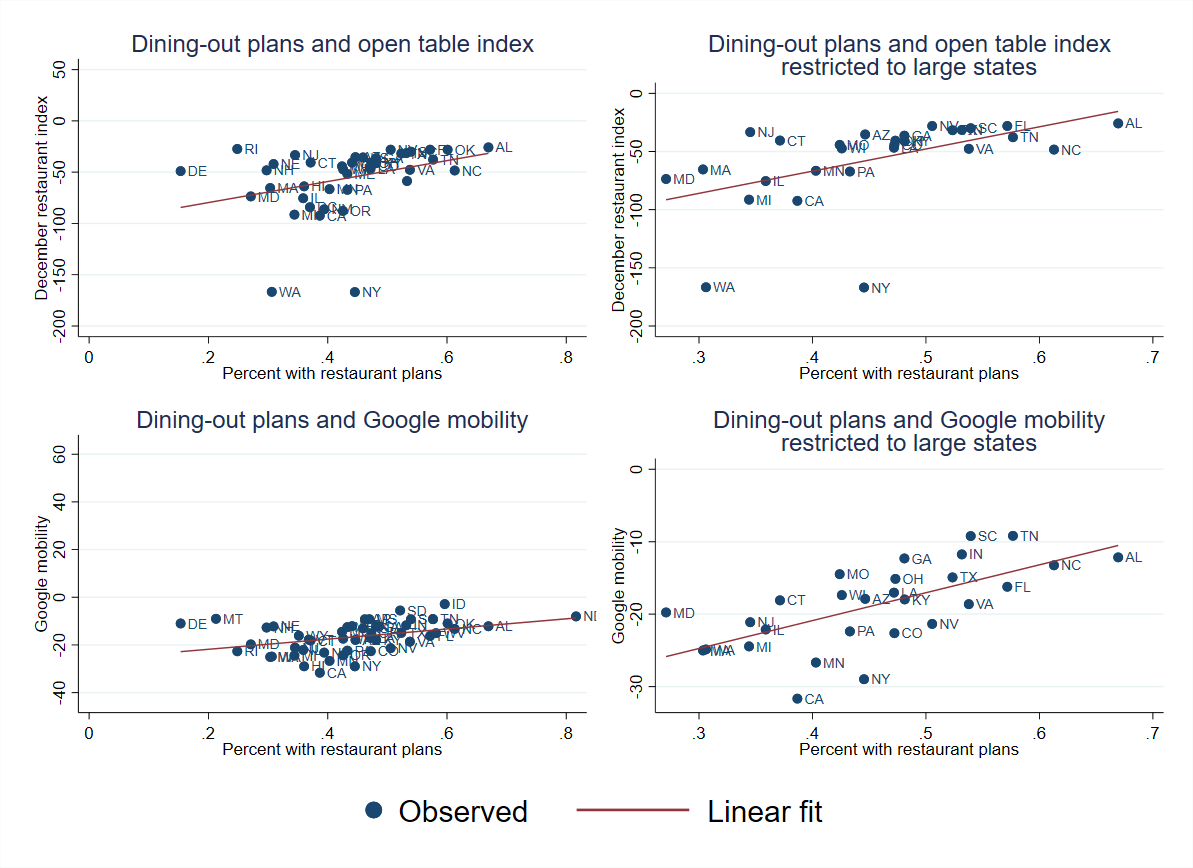


References

Cokely, E., Galesic, M., Schulz, E., Ghazal, S., and Garcia-Retamero, R. (2012). Measuring risk literacy: the berlin numeracy test. Judgment and Decision Making, 7(1):25–47.

Stigler, G. J. (1961). The economics of information. Journal of Political Economy, 69(3):213–225

1. OpenTable, data accessed March 30, 2022 via <https://www.opentable.com/state-of-industry>. Google Mobility data, accessed August 1, 2021, via <https://www.google.com/covid19/mobility/> [↑](#footnote-ref-1)
